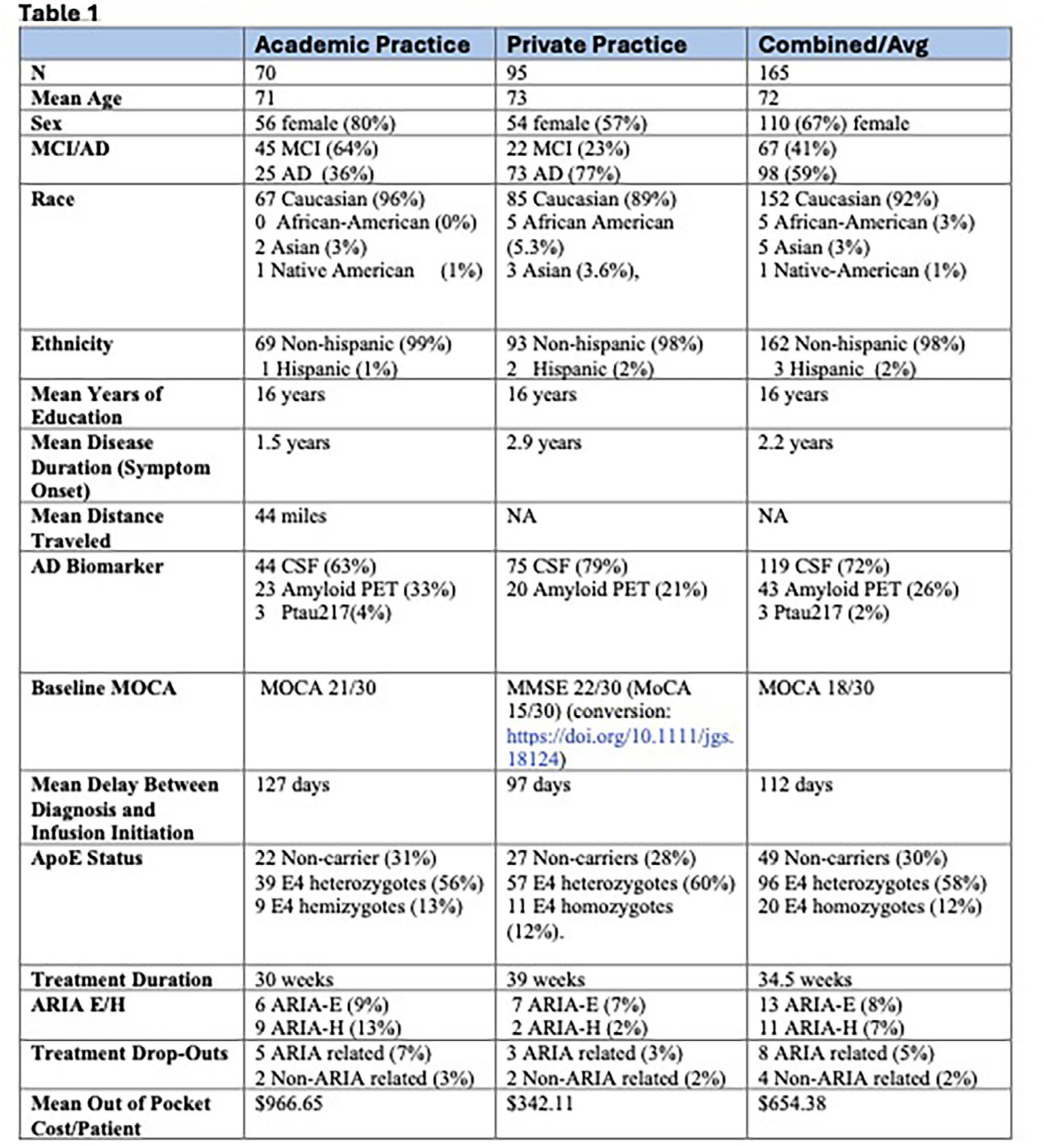# Monoclonal Antibody Administration in an Academic Institution and Private Neurological Practice: A Tale of Two Clinics

**DOI:** 10.1002/alz70861_108011

**Published:** 2025-12-23

**Authors:** David C Weisman, Michael Henry Rosenbloom

**Affiliations:** ^1^ Abington Neurologic Associates, Abington, PA USA; ^2^ Memory and Brain Wellness Center, University of Washington, Seattle, WA USA

## Abstract

**Background:**

The emergence of anti‐amyloid monoclonal antibody (MABs) drugs since the FDA approval of lecanemab has resulted in dramatic changes in the clinical approach and management of early‐stage Alzheimer’s disease (AD). Challenges with MAB adoption into clinical practice may vary depending on whether the institution is an academic/integrated healthcare organization versus a private neurological practice.

**Method:**

We combined demographic and clinical data from a high‐volume East coast private neurology practice and a West coast academic memory clinic at post‐MAB adoption.

**Result:**

Combined data of N=165 patients showed the following demographics: mean age 72, 67% female, 92% Caucasian, average MOCA 18/30 with amyloid status confirmed by CSF in 72% of patients. Overall, ARIA rates were 8% for ARIA‐E and 7% for ARIA‐H, and there were no mortalities over the one‐year period. Two patients required immediate medical attention due to severe ARIA radiographic findings associated with clinical symptoms. The private practice enrolled patients with lower MOCA scores than the academic practice (15 versus 22), but was more efficient at initiation therapy (mean # of weeks between diagnosis and treatment 20 versus 26 weeks). The average patient out of pocket cost was ($654.38) significantly less than the 20% of the annual drug cost as previously estimated.

**Conclusion:**

The findings from two separate clinical environments support the notion that ARIA risk associated with lecanemab is no greater than what was found in the CLARITY‐AD trial and that the costs to the patient were less than predicted. Additional real‐world data relating to the clinical effectiveness of MAB use in clinical practice will be necessary to best determine the risk/benefit ratio of these drugs in community populations.